# Text data extraction for a prospective, research-focused data mart: implementation and validation

**DOI:** 10.1186/1472-6947-12-106

**Published:** 2012-09-13

**Authors:** Monique Hinchcliff, Eric Just, Sofia Podlusky, John Varga, Rowland W Chang, Warren A Kibbe

**Affiliations:** 1 Department of Medicine, Division of Rheumatology, Northwestern University Feinberg School of Medicine, Chicago, USA; 2 Northwestern Medical Enterprise Data Warehouse, Chicago, USA; 3 Department of Preventive Medicine, Northwestern University Feinberg School of Medicine, Chicago, USA; 4 Physical Medicine and Rehabilitation, Northwestern University Feinberg School of Medicine, Chicago, USA; 5 Robert H. Lurie Comprehensive Cancer Center, Northwestern University Biomedical Informatics Center, Chicago, USA; 6 Northwestern University Feinberg School of Medicine, McGaw Pavilion, Suite M300, 240 E Huron Street, Chicago, IL, 60611, USA

**Keywords:** Medical informatics, Information storage and retrieval, Information systems, Electronic health records, Automatic data processing

## Abstract

**Background:**

Translational research typically requires data abstracted from medical records as well as data collected specifically for research. Unfortunately, many data within electronic health records are represented as text that is not amenable to aggregation for analyses. We present a scalable open source SQL Server Integration Services package, called Regextractor, for including regular expression parsers into a classic extract, transform, and load workflow. We have used Regextractor to abstract discrete data from textual reports from a number of ‘machine generated’ sources. To validate this package, we created a pulmonary function test data mart and analyzed the quality of the data mart versus manual chart review.

**Methods:**

Eleven variables from pulmonary function tests performed closest to the initial clinical evaluation date were studied for 100 randomly selected subjects with scleroderma. One research assistant manually reviewed, abstracted, and entered relevant data into a database. Correlation with data obtained from the automated pulmonary function test data mart within the Northwestern Medical Enterprise Data Warehouse was determined.

**Results:**

There was a near perfect (99.5%) agreement between results generated from the Regextractor package and those obtained via manual chart abstraction. The pulmonary function test data mart has been used subsequently to monitor disease progression of patients in the Northwestern Scleroderma Registry. In addition to the pulmonary function test example presented in this manuscript, the Regextractor package has been used to create cardiac catheterization and echocardiography data marts. The Regextractor package was released as open source software in October 2009 and has been downloaded 552 times as of 6/1/2012.

**Conclusions:**

Collaboration between clinical researchers and biomedical informatics experts enabled the development and validation of a tool (Regextractor) to parse, abstract and assemble structured data from text data contained in the electronic health record. Regextractor has been successfully used to create additional data marts in other medical domains and is available to the public.

## Background

Translational research requires the longitudinal collection of anthropometric, demographic, laboratory, and diagnostic data for specific patient cohorts. Most institutions now have electronic health record systems to collect and retain these data. Electronic health records are often document-centered systems [1] that require special processing [2-4] to collect and aggregate data, especially when clinical data is stored as text (e.g. clinical notes, diagnostic studies such as pulmonary function tests, and some quantitative laboratory results). Electronic health records enable the examination of these data on a per-encounter or per-patient basis, but are not designed to enable the aggregation of data across a series of encounters or for a defined cohort of patients, as is typically required in clinical research. Lack of aggregated clinical data necessitates costly and error-prone manual chart reviews [5]. There are several technical projects designed to aid in transforming clinical text into discrete, analyzable data. These projects are typically bundled as large frameworks requiring additional servers and computing environments to implement [2,6-8].

Data warehouses that are large databases used for reporting and analyses, greatly facilitate data aggregation by integrating data from a number of sources, including electronic health records. Data warehouses provide a consistent, defined access mechanism to electronic health record data through standard database reporting and analytics tools. Data warehouses are often used to spawn specialized data marts that contain pre-processed or transformed versions of specific cross-sections of data available in the warehouse. Coupling a data mart to a data warehouse through one or more structured and reproducible transformation processes allows data warehouse architects to easily maintain a well defined data source while providing clinical researchers with necessary clinical research data.

### Case description-technical environment

The Northwestern Medical Enterprise Data Warehouse (Medical Enterprise Data Warehouse) is a 10 TB electronic Microsoft SQL Server 2008R2 database developed to collect and integrate patient information obtained from over 30 medical and clinical research database systems deployed across the medical campus. The campus includes in- and out-patient facilities and the Feinberg School of Medicine. The Medical Enterprise Data Warehouse contains copies of many of these database systems, also known as operational data stores in a single database and integrates the data via a campus-wide patient identifier. Data marts typically join data from several operational data stores tables to provide enterprise data warehouse query writers with integrated, aggregated, or processed data.

Microsoft SQL Server Integration Services is the extract, transform and load tool used to create both operational data stores and data marts within the Medical Enterprise Data Warehouse. A developer defines a source query in an external system, adds one or more transformations that manipulate data from a source query, and writes the results of the transformations to tables within the Medical Enterprise Data Warehouse. The most common transformations in a data flow include type casting, adding columns to a data flow, or applying logical operators to data. SQL Server Integration Services comes with a number of pre-defined transformation components, but provides an Application Programmer Interface that allows programmers to create their own custom components. In academic medical centers, recent surveys show the use of Microsoft SQL Server for data warehousing to approach or exceed 50% [9].

### Pulmonary function test data flow

**Figure 1:**
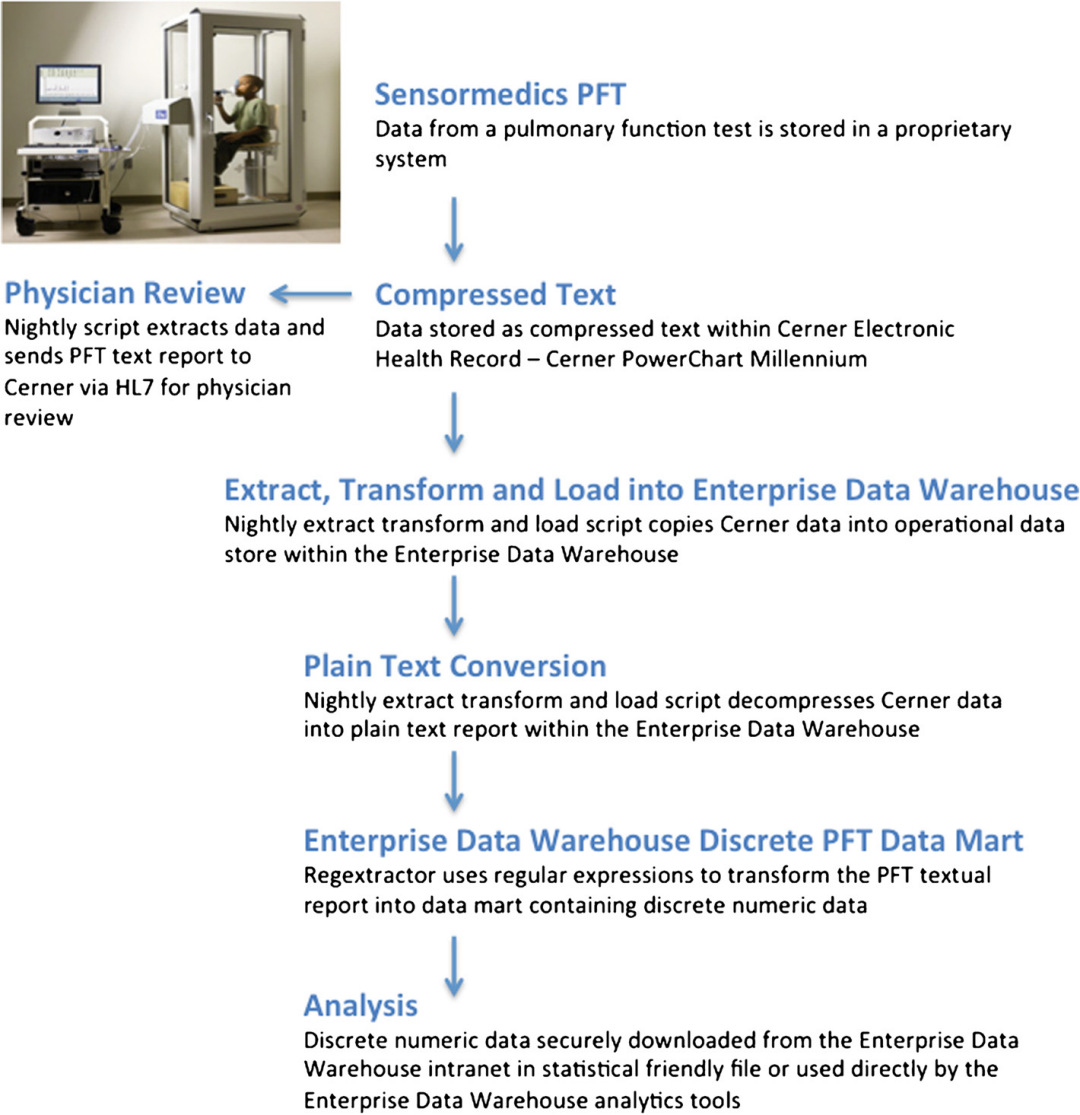
PFT data flow pipeline from the pulmonary function test laboratory to the clinician and physician/researcher.

Pulmonary function tests (PFT) are a group of tests (spirometry, lung volumes and diffusion capacity for carbon monoxide) that assess how well a patient moves air in and out of the lungs and how easily inhaled gas moves from the lungs into the blood stream (see Additional file 1: Figure S1 to view an actual pulmonary function test report and Table 1 for a list of discrete data elements captured by PFT instrumentation). A patient undergoing PFT is seated in a chamber such as the SensorMedics Vmax Encore PFT Autobox Pro machine while they repeatedly perform deep inhalations and exhalations (Figure 1). Pulmonary function data is captured using SensorMedics software version IVS-0101-21-1A and stored in a proprietary database used by the SensorMedics software (CareFusion Corporation, San Diego, CA). A nightly script extracts data from the Sensor Medics database to generate a common clinical text report (Additional file 1: Figure S1). The report is sent via HL7 messaging for inclusion in the Cerner Millennium electronic health record. On a nightly basis, the Medical Enterprise Data Warehouse is synchronized with the Cerner Millennium Oracle database. Because text data within Cerner is stored in a proprietary binary format, special processing is required for deserialization. Medical Enterprise Data Warehouse data architects created a plain text data mart consisting of active clinical texts from Cerner Millennium including textual PFT data. To facilitate the aggregation of discrete numeric data from text reports, we developed an open source text extraction SQL Server Integration Services package called Regextractor. We present this package and the results of a validation study that confirms the value and consistency of this approach.

**Table 1:** Description of data elements available in PFT data mart

		**Measures**
Parsed Numeric Columns	Spirometry (measures the airflow in and out of the lungs)	54
	Lung volumes (assess the volume of air associated with different phases of the breath)	26
	Diffusing Capacity for Carbon Monoxide (measures the ease with which a test gas (carbon monoxide) crosses the air sac membrane into the blood stream)	12
	Pulse Oximetry (assesses the oxygenation of hemoglobin)	2
	Anthropometric (height and weight)	2
Parsed Non-numeric Columns	Non-numeric data about patient (race, ethnicity, ordering physician, notes)	6
	Data about measurements (date, technician, etc.)	5
**Totals**	**Total number of potential measures parsed into discrete data elements**	**107**

Not all patients undergo all the components of each PFT so the PFT report within the electronic health record may contain fewer measures (Additional file 1: Figure S1).

## Methods

### Creation of the PFT Data Mart

SQL Server Integration Services does not include a transformation component to facilitate use of regular expressions to extract data from a text field. In order to parse structured PFT text data in the SQL Server Integration Services extract transform and load platform, we created a custom component called Regextractor. We wrote a PFT extract transform and load package consisting of a query to pull PFTs followed by a series of Regextractor components parsing the textual data using 39 regular expressions (Additional file 2: Figure S2). These expressions parse 107 data fields with some of the regular expressions parsing multiple data fields. The regular expressions match specific patterns of data in the text reports, accommodating known variation in the report format. For instance, some PFT components are not administered to all patients.

### Description of the Pulmonary Function Test Data Mart

The PFT data mart contained 43,364 PFTs as of 6/26/2012 and has 107 measures for each PFT (Table 1). Ninety-six data elements are discrete numeric measurements. Eleven data elements are non-numeric fields such as name, patient type (inpatient/outpatient), and date. Six columns are keys to other tables in the Medical Enterprise Data Warehouse to facilitate integration with other data marts such as the master patient index, the encounter data mart, and orders. For instance, the encntr_id field links to the main encounter data mart where admit, discharge, and some billing information is stored.

### Validation

Patients with systemic sclerosis/scleroderma, a rare connective tissue disease that causes skin and internal organ fibrosis especially in the lungs, vascular disease, and autoantibody production, undergo PFTs to screen for the development and progression of lung disease. Scleroderma predominately affects middle-aged women, and lung disease is the leading cause of death [10]. As a result, aggregate PFT data are required for many scleroderma translational research projects and provide a measure of disease progression in patients with scleroderma. In the past, scleroderma research assistants printed PFT reports and manually entered data into data capture tools such as spreadsheets. Because this process was time consuming, ecologically unfriendly and error prone, a new system was developed in conjunction with Medical Enterprise Data Warehouse data architects to generate a PFT data mart within the Warehouse that can readily be queried and maintained through automated processes.

To validate the integrity of data within the PFT data mart, 100 subjects from a cohort >500 patients in the Northwestern Scleroderma Registry were randomly selected. All participants met the American College of Rheumatology criteria for scleroderma and had consented to partake in medical research [11]. One PFT per participant was chosen, with the test performed closest to the date of consent to the registry selected for inclusion in the analysis (12/2004-9/2010). Eleven data elements (forced vital capacity and forced vital capacity% predicted from spirometry tests; total lung capacity, total lung capacity% predicted from lung volume tests; and diffusion capacity for carbon monoxide and diffusion capacity for carbon monoxide% predicted from diffusion tests, as well as medical record number, height, weight, gender, and exam date) were selected for each research participant (Additional file 1: Figure S1 and Table 1). Medical record number, and exam date were required in order to conduct the validation study with manual chart review. Clinically relevant variables included three anthropometric variables (height, weight and gender) that directly influence pulmonary function, as well as six clinically relevant discrete pulmonary function parameters (forced vital capacity, forced vital capacity% predicted; total lung capacity, total lung capacity% predicted, diffusion capacity for carbon monoxide and diffusion capacity for carbon monoxide% predicted.

For the manual chart abstraction, a scleroderma research assistant printed the PFT report and manually entered relevant data into a Research Electronic Data Capture (REDCap) database housed at Northwestern [12]. To ensure an error rate < 5%, the same research assistant reentered the data into the database one week after the initial entry. From the automated data mart, Medical Enterprise Data Warehouse report writers generated a PFT report for the same patient cohort that was securely delivered using SQL Server Reporting Services. Researchers downloaded these data through the Medical Enterprise Data Warehouse intranet web portal in a statistics package friendly format (comma separated values). Statistical analyses to determine the correlation between data obtained using the automated and manual abstraction methods were conducted using STATA version 10.1 (College Station, TX).

## Results

The initial PFT data mart was built by coupling Regextractor to the existing extract transform and load process using SQL Server Integration Services. Reports pulled from the resulting PFT data mart were validated against manual chart abstraction. There were six discrepancies out of 1100 (99.5% congruency) between the results obtained from the manual chart abstraction process and the PFT data mart. In all cases, the discrepancies were due to mistakes made during data entry.

## Discussion

The abstraction and aggregation of clinical data from electronic health records is a necessary component of translational research. We have presented a way to abrogate the need for researchers to re-enter numerical data available in structured reports in clinical systems. We recognize that regular expressions are only appropriate for highly structured, machine-produced data and span the gap between structured data entry and natural language processing techniques for semantically interpreting text such as clinical notes. Based on other work, about 40% of the data elements involved in translational research are captured in electronic health records at various levels of structure, so this process is of general utility [13-15]. Automating the abstraction process and representing structured textual data as discrete, atomically coded values streamlines the availability of these data for translational research and outcomes analysis. We have designed a reusable SQL Server Integration Services package (Regextractor) for extracting coded data from text fields in the electronic health record and demonstrated the creation of a discretely coded PFT data mart using Regextractor. Regextractor has also been used to create cardiac catheterization and echocardiographic data marts at our institution [16].

### Clinical utility and generalizability of the PFT Data Mart

Longitudinal analysis of PFT results provides important outcome measures for scleroderma and other disease areas and research activities involving pulmonary function. The automated system described in this paper was created to populate a structured PFT data mart without requiring double data entry or manual chart review. To enable future reuse of the PFT data mart, all data elements and data points from PFT reports beyond those required for the validation study were parsed and abstracted into the data mart. While this comprehensive approach lengthened the time to deliver the initial PFT data, the resulting data mart facilitated the use of the data mart for other research. For instance, scleroderma investigators have used the PFT data mart to build six additional queries, and three other Northwestern researchers utilized the PFT data mart for other studies [17]. Although the requirement for a consistent format of the SensorMedic PFT data would appear to be a limitation, changes in the format of the underlying electronic health record data requires substantial revision of many parts of the extract, transform and load process. In this particular case, the PFT data format has remained constant for >10y, but changes in the format or the data element definitions would require revision to the extract, transform, load process as well as the regular expressions.

### Technical generalizability of text processing workflow

Prior to the PFT data mart, there were no processed text data marts in the Northwestern Medical Enterprise Data Warehouse. Before Regextractor, the only way to embed regular expressions into the SQL Server Integration Services extract, transform and load process was to custom build and compile a new SQL Server Integration Service package. The overhead of the compile and testing cycle made the incorporation of regular expression parsing into the extract, transform and load process tedious at best. The ‘Regextractor’ SQL Server Integration Services package makes it easy to modify the regular expressions without recompiling and incorporates the parsing pipeline into existing extract, transform and load processes. Although the Regextractor package was developed to parse PFT reports for the Scleroderma Registry, it provides a general tool for incorporating regular expression parsing into any SQL Server Integration Services-orchestrated extract transform and load process.

We have released the Regextractor text-parsing package as open-source software. The project is hosted on Codeplex, an open source hosting site and the code is available at http://regextractor.codeplex.com. The component is easy to integrate into existing extract transform and load processes such as those used to build enterprise data warehouses and have been downloaded 552 times as of 6/1/2012. Also, the regular expressions to parse the PFT reports are highly transportable to common scripting languages such as Perl, Python and Ruby.

### Accuracy and efficiency of electronically populated data marts

Our results demonstrate that the automated abstraction of numerical data from structured text was 100% reproducible for 100 PFT cases. In contrast, double entry manual chart review resulted in six errors for 1100 data points entered (0.5% error rate). The manual data extractor made typographical errors that accounted for five of the six inconsistencies. These errors resulted in two inconsistent medical record numbers, two incorrect test dates, and one inconsistency in height. The final error was due to incorrect coding for gender during manual data entry: female was entered instead of male. The 0.5% manual data entry error rate is well below the reported 10% error rate for manual data extraction using a paper intermediate [5], but still underscores the importance of designing systems to electronically collect and integrate research data and the benefit of automatic data abstraction whenever feasible. Just as importantly, the manual abstraction scales linearly with the number of PFTs being analyzed, whereas the automated data mart approach has scaled to tens of thousands of records and is nearly independent of the number of PFT records analyzed. The manual error rate was low for several reasons – double entry typically reduces the data error rate significantly, the abstraction was done with case histories and patient information available, and the chart abstractor was familiar with PFT data.

## Conclusions

The results presented here demonstrate how collaboration between data warehouse architects and clinical researchers created a process for the establishment of data marts that facilitated access to electronic health record data for research at our institution. We have built a general tool (Regextractor) and developed a methodology for parsing, abstracting and assembling structured data from textual instrument data in our electronic health record. We have applied this methodology and demonstrated the use of this tool in creating a multiuse PFT data mart. We have also shown that this approach resulted in an accurate PFT data mart, defined a validation approach for the PFT, used Regextractor to create additional data marts in other medical domains, and demonstrated that the use of Regextractor is more efficient and scalable than manual chart abstraction.

## Abbreviation

PFT: Pulmonary function test.

## Competing interests

No financial support or other benefits from commercial sources for the work in the manuscript or any other financial interest that any of the authors have, create a conflict of interest with regard to the work.

## Authors’ contributions

MH conceived and designed the study, interpreted the data and co-drafted the manuscript. EJ created Regextractor, interpreted the data, and co-drafted the manuscript. SP participated in the design of the study and performed manual chart abstraction. JV influenced study design and data interpretation. RWC assisted in study design, data interpretation, and helped to draft and edit the manuscript. WK assisted in study design, data interpretation, helped to draft and edit the manuscript. All authors read and approved the final manuscript.

## Authors’ information

MH is an assistant professor of medicine in the Northwestern University Feinberg School of Medicine and the Associate Clinical Director of the Northwestern University Scleroderma Program and played a pivotal role in the establishment of the Northwestern Scleroderma Program Patient Registry and Biorepository in 2008. MH enlists the support of bioinformatics and enterprise data warehouse experts (EJ and WAK) at the Northwestern Medical Enterprise Data Warehouse and the Northwestern University Bioinformatics Center (NUBIC) to create automated data capture systems to ensure the integrity of clinical data collected for scleroderma research. WAK is a professor of biomedical informatics in the Northwestern University Feinberg School of Medicine. His research interests include the use of open biomedical ontologies to accelerate translational research and the application of agile software practices to biomedical research software solutions.

## Pre-publication history

The pre-publication history for this paper can be accessed here:

http://www.biomedcentral.com/1472-6947/12/106/prepub

## Supplementary Material

Additional file 1 **Figure S1.** A de-identified PFT report that is the source of the measures in the PFT data mart. Click here for file

Additional file 2 **Figure S2.** Series of 39 regular Regextractor expressions used to PFT textual data. Click here for file
