# Supplementary material for: Text data extraction for a prospective, research-focused data mart: implementation and validation
Source: BMC Med Inform Decis Mak. 2012 Sep 13;12:106. doi: 10.1186/1472-6947-12-106 (PMC3537747; doi:10.1186/1472-6947-12-106)
Supplement: Additional file 1 — Figure S1. A de-identified PFT report that is the source of the measures in the PFT data mart. [file 1472-6947-12-106-S1.doc]

Figure 3
