# Supplementary figures and images for: Text data extraction for a prospective, research-focused data mart: implementation and validation
Source: BMC Med Inform Decis Mak. 2012 Sep 13;12:106. doi: 10.1186/1472-6947-12-106 (PMC3537747; doi:10.1186/1472-6947-12-106)

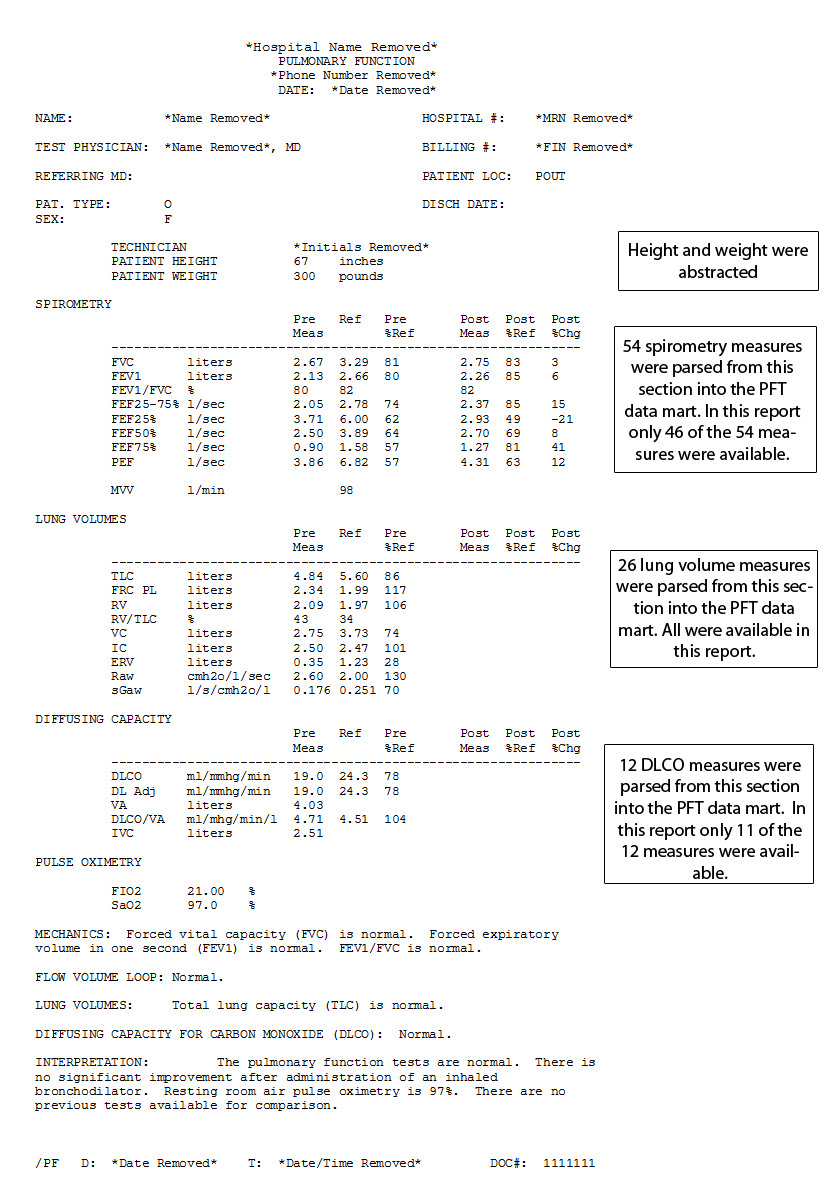

Supplement: Additional file 2 — Figure S2. Series of 39 regular Regextractor expressions used to PFT textual data. [file 1472-6947-12-106-S2.tiff]
